# Scaling law to determine peak forces in tapping-mode AFM experiments on finite elastic soft matter systems

**DOI:** 10.3762/bjnano.8.98

**Published:** 2017-05-02

**Authors:** Horacio V Guzman

**Affiliations:** 1 Max Planck Institute for Polymer Research, Ackermannweg 10, 55128 Mainz, Germany

**Keywords:** AFM in liquid, AFM theory, bidimensional elastic models, multivariate regression, neuronal networks, operational AFM parameters, parametrical equation, peak forces, soft matter

## Abstract

Analytical equations to estimate the peak force will facilitate the interpretation and the planning of amplitude-modulation force microscopy (tapping mode) experiments. A closed-form analytical equation to estimate the tip–sample peak forces while imaging soft materials in liquid environment and within an elastic deformation regime has been deduced. We have combined a multivariate regression method with input from the virial–dissipation equations and Tatara’s bidimensional deformation contact mechanics model. The equation enables to estimate the peak force based on the tapping mode observables, probe characteristics and the material properties of the sample. The accuracy of the equation has been verified by comparing it to numerical simulations for the archetypical operating conditions to image soft matter with high spatial resolution in tapping-mode AFM.

## Introduction

Amplitude-modulation atomic force microscopy (AM-AFM) is the most common method to generate atomic and molecular resolution images of diverse materials in liquid environment [1–15]. In AM-AFM (tapping mode) a sharp tip is attached at the end of a microcantilever that oscillates at its fundamental flexural resonant frequency while the amplitude is used as the feedback parameter to record the topography while imaging. When the tip is in close proximity to the sample the amplitude and the phase shift of the oscillation change with the strength of the tip–sample interaction forces. To image soft matter without generating plastic deformations, it is necessary to determine beforehand the force exerted to the sample. However, the force is not a direct observable in AM-AFM. The force-inversion methods offer an alternative but these methods provide the force estimation on an a posteriori basis*.* Moreover, those methods could be very time consuming to tune for non-expert enthusiastic AFM experimentalists and their accuracy is under debate within the dynamic AFM community [16–17].

Numerical simulations and analytical scaling laws are well-established methods to estimate the interaction forces of a measurement [9,18–26]. One of the latter methods is the estimation of the peak interaction forces [21–23,25–29]. The peak interaction force determines the deformation on the sample and hence the spatial resolution and the degree of invasiveness of the measurement. The parametrical equation obtained by Raman et al. has been based on the Hertzian mechanics for air and vacuum environments [21]. It has been also adapted to estimate the experimental peak forces of viral capsids (ca. 1 GPa) in liquid [22]. However, those parametrical approximations have not been designed to describe the forces for finite soft-matter systems in highly damping environments. In this article we use the term soft matter to describe polymeric surfaces and/or biological systems (isolated or packed arrays of proteins) with Young moduli in the range of 30–300 MPa [11–12,14,30–31]. Moreover, we provide the explicit method to obtain an analytical equation based on the relevant dynamic AFM operational parameters.

Here, a parametrical equation to determine the peak interaction force exerted by the AM-AFM on a finite soft material immersed in a liquid environment has been deduced. Such deduction has been based in the previous works by the author [15]. The fact of reducing the electrostatic interactions of the surface charge depends strongly on the ionic strength and pH values of the liquid [32]. Thus, immersing the surface sample and the probe into a liquid with a certain salt concentration that reduces the electrostatic interactions to a minimum are assumed as medium conditions in this article [33]. The use of Hertzian mechanics has been generalized to model the tip–sample interaction forces for relatively rigid materials [34]. However, for finite soft matter Tatara’s contact mechanics model could be more appropriate to describe the elastic interactions between tip and sample. In particular when the sample is very soft and has finite dimensions conditions that would imply that the deformation happens symmetrically at both, the tip–sample and the sample–substrate interfaces [35–37].

## Results and Discussion

### Equation of motion and tip–sample forces

The dynamics of the microcantilever–tip system in AM-AFM can be approximated by the second-order non-linear differential equation [38],

[1]
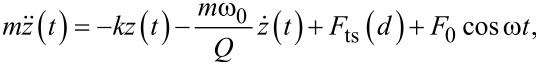


where *m* is the effective microcantilever–tip mass that includes the added mass of the fluid [39], and ω_0_, *Q*, *k* and *F*_ts_ are the angular resonant frequency, quality factor, spring constant and tip–sample interaction forces, respectively. The latter has been modelled according to the Tatara contact mechanics [35–37] which is given by

[2]
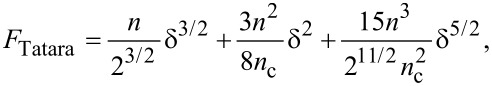


with the constitutive material variables

[3]
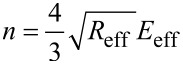


and

[4]
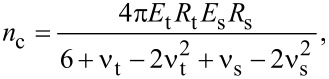


where the indexes “t” and “s” stand for tip and sample, respectively, in the above equations, δ is the indentation, ν is the Poisson coefficient (ν_t_ = 0.3 and ν_s_ = 0.4) and *E* is the Young’s modulus with *E*_t_ = 170 GPa. The effective Young’s modulus *E*_eff_ and radius *R*_eff_ are described elsewhere [28–29].

### Multivariate regression method to find a parametrical equation for the peak forces in tapping mode AFM on finite elastic soft matter systems

Asymptotic approximation methods have been used to deduce parametrical equations of physical quantities in dynamic systems. In amplitude-modulation AFM these theoretical approximations have been applied to derive a parametrical equation for determining the peak force based on the addition of repulsive Hertzian and attractive van der Waals interactions in low-damping environments [21]. Here we have conceived a multivariate regression analysis to obtain a parametrical equation of the peak interaction forces according to a bidimensional elastic contact mechanics model, namely Tatara’s one (see Equation 2). The main method's assumption is that the peak interaction force can be expressed as a sixth-order multivariate cascade function [40–41] of the aggregated AFM parameters,

[5]
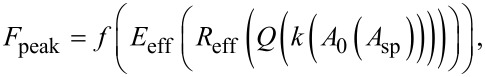


where *A*_sp_ is the set-point amplitude. Other variables have been previously described in Equation 1 and Equation 3. Equation 5 reflects a highly complex function relating the independent operational, probe and materials properties variables of the intrinsic nonlinear system (AM-AFM).

**Table 1:** Simulation parameters defined for the multivariate regression analysis. In all cases the values of the ratio between tip and sample are *R*_s_ = 0.8*R*_t_ and *f*_0_ = 25 kHz.

parameters	*E*_s_ (MPa)	*A*_0_ (nm)	*k* (N/m)	*A*_sp_	*Q*	*R*_t_ (nm)

range	30–300	1–10	0.1–1.0	(0.7–0.95)*A*_0_	1–5	5, 7.5, 10
steps	270	20	10	6	5	—

One strategy to reduce the order of the cascade function (Equation 5) is to base our modelling on top of one available analytical approach to determine *F*_ts_ in dynamic AFM [21,42–43]. We have applied the virial–dissipation method [19] to determine an initial equation for the peak force as a function of the relevant amplitudes. Hence, Equation 5 is reduced to

[6]
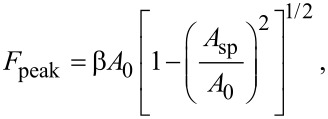


where


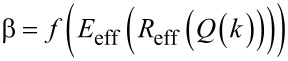


is a force coefficient that depends on the four aggregated AFM variables *E*_eff_, *R*_eff_, *Q*, and *k*. The multivariate regression is a systematic method to perform regressions in a certain given sequence. Such method applied to β begins by building a one-variable regression of *k* then continues with *Q*, *R*_eff_ and finally *E*_eff_. These multiple regressions were based on the numerical simulations results for the operational, probe and materials properties values given in Table 1, letting,

[7]
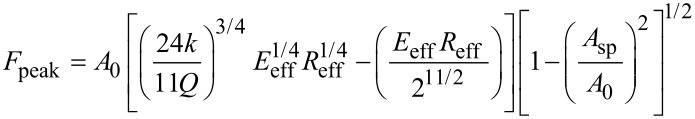


with a cumulative coefficient of determination of *R*^2^ ≈ 0.85, which is acceptable for a sixth-order multivariate regression analysis. However, such coefficient of determination has been applied to a fourth-order multivariate regression only. Hence, by applying a loop of regressions while maximizing *R*^2^ based on the power variable *x* in the expression [1 − (*A*_sp_/*A*_0_)^2^]*^x^*. The cumulative coefficient of determination can be enhanced to *R*^2^ ≈ 0.91 and Equation 7 becomes

[8]
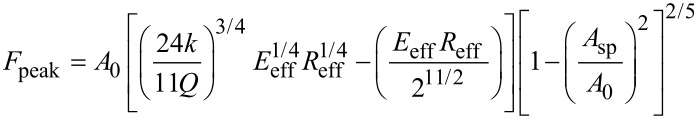


### Dependence of the peak forces parametrical equation on the samples Young’s moduli

To verify the theoretical predictions for *F*_peak_ (Equation 8), we have compared them to numerical simulations for the operating conditions needed to image soft samples with high spatial resolution [28–29]. Whereby the peak forces are minimized by using small free amplitudes of 1 to 4 nm and soft cantilevers with *k* = 0.1 N/m. Note that those parameters are constrained to the range of operational, probe and materials properties described in Table 1. Nonetheless, this range could be extended for some probe and/or operational parameters such as *k*, A_0_ and/or *R*_t_. However, for such extensions a new cumulative correlation coefficient must be obtained.

**Figure 1:**
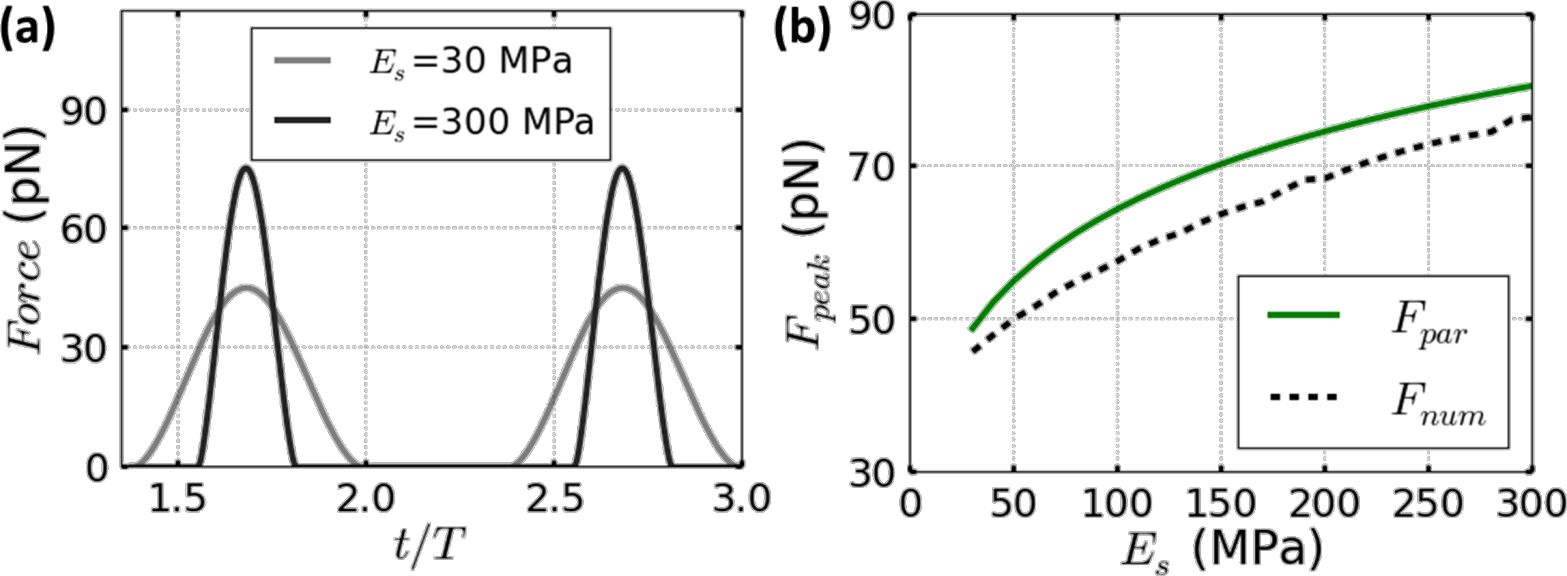
(a) The dependence of the forces on the normalized time for two different Young moduli 30 MPa (light gray line) and 300 MPa (dark gray line). (b) The dependence of the peak forces on the sample Young modulus for the parametrical equation of Equation 8 (full line plots) and the corresponding numerical simulations for Tatara’s contact mechanics (dashed line plots). Parameters defined for (a) and (b) are: *A*_0_ = 1 nm, *A*_sp_ = 0.9*A*_0_, *Q* = 2, *k* = 0.1 N/m and *R*_t_ = 5 nm.

In Figure 1a, the simulated force behavior as a function of the time is shown for two different materials with Young’s moduli of 30 and 300 MPa, respectively. For each material the peak force is defined as the maximum repulsive interaction of the time domain curves observed in Figure 1a. We quantitatively explored through simulations how the contact time increases with the lower Young’s modulus values of the material. Figure 1b shows the comparison of the parametrical equation and numerical simulations for the whole range of Young moduli between 30 and 300 MPa for *A*_sp_ = 0.9*A*_0_.

**Figure 2:**
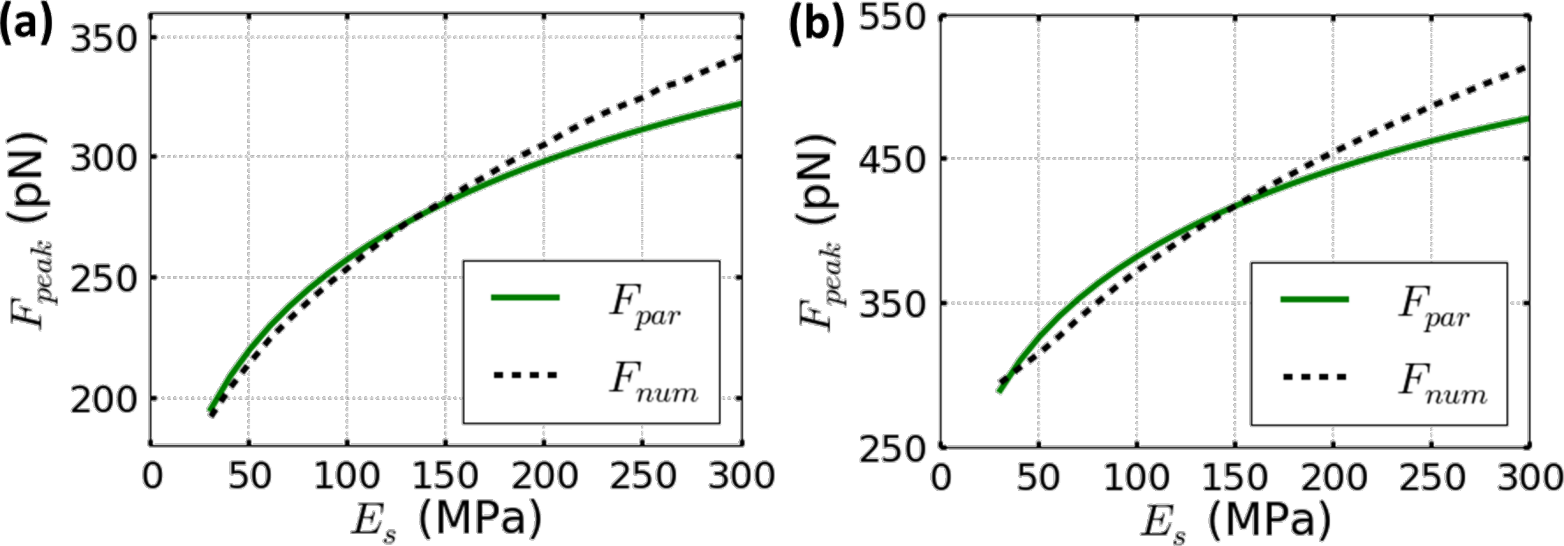
The dependence of the peak forces on the sample Young’s modulus for the parametrical equation of Equation 8 (full line plots) and the corresponding numerical simulations for Tatara’s contact mechanics model (dashed line plots). (a) *A*_sp_ = 0.9*A*_0_ and (b) *A*_sp_ = 0.7*A*_0_. Parameters defined for (a) and (b) are: *A*_0_ = 4 nm, *Q* = 2, *k* = 0.1 N/m and *R*_t_ = 5 nm.

Figure 1b and Figure 2 compare the parametrical equation of Equation 8 and the corresponding numerical simulations for Tatara’s contact mechanics model. The comparisons cover Young’s moduli in the range of 30 to 300 MPa, and two fixed set-point amplitudes, namely 0.9*A*_0_ and 0.7*A*_0_. In addition, the spring constant is fixed to *k* = 0.1 N/m and the free oscillation amplitudes are 1 nm (Figure 1b) and 4 nm (Figure 2). The peak force increases monotonically with the Young’s modulus of the sample. These results are consistent with previous numerical simulations [28–29].

In Figure 1b, the agreement between the parametrical equation and the numerical simulations in the explored range remains close to a maximum relative error of 10%. This value can be also considered as a worst case scenario in the a priori estimation of the applied forces to soft matter. In Figure 2, a similar comparison is performed but for a free amplitude *A*_0_ = 4 nm. The accuracy of the parametrical equation shown for both cases is fully within the 10% of relative error. However, the relative error is slightly increased by decreasing the set-point amplitude value from 0.9*A*_0_ (Figure 2a) to 0.7*A*_0_ (Figure 2b). The dependence of the relative error on the set-point amplitude has been previously argued about in another peak force parametrical scaling law which is also based on asymptotical approximations [21–22].

### Dependence of the peak forces parametrical equation on the set point amplitudes

**Figure 3:**
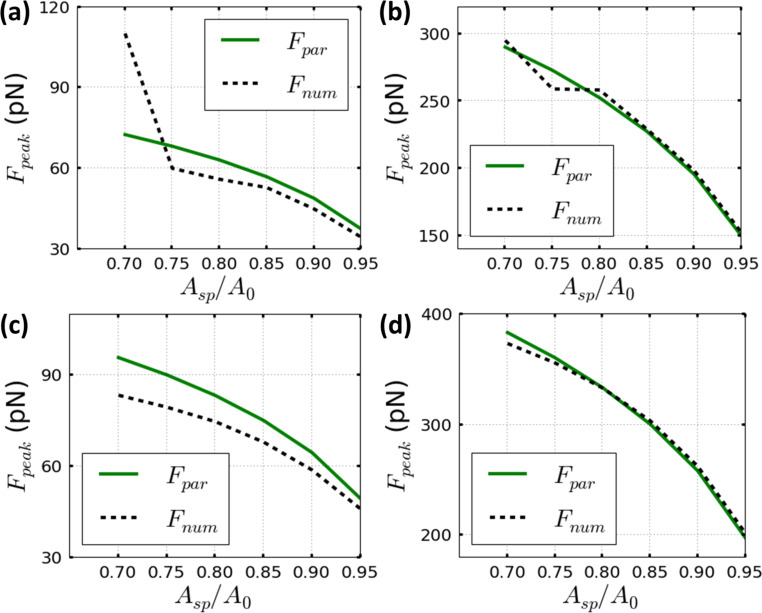
The dependence of the peak forces on the set-point amplitude for the parametrical equation of Equation 8 (full line plots) and the corresponding numerical simulations for Tatara’s contact mechanics (dashed line plots). (a) Material with a Young’s modulus of 30 MPa and *A*_0_ = 1 nm. (b) Material with the same Young's modulus as (a) and a higher free amplitude *A*_0_ = 4 nm. (c) Material with a Young’s modulus of 100 MPa and *A*_0_ = 1 nm. (d) Material with the same Young’s modulus as (c) and a higher free amplitude *A*_0_ = 4 nm. Parameters are: *Q* = 2, *k* = 0.1 N/m and *R*_t_ = 5 nm.

**Figure 4:**
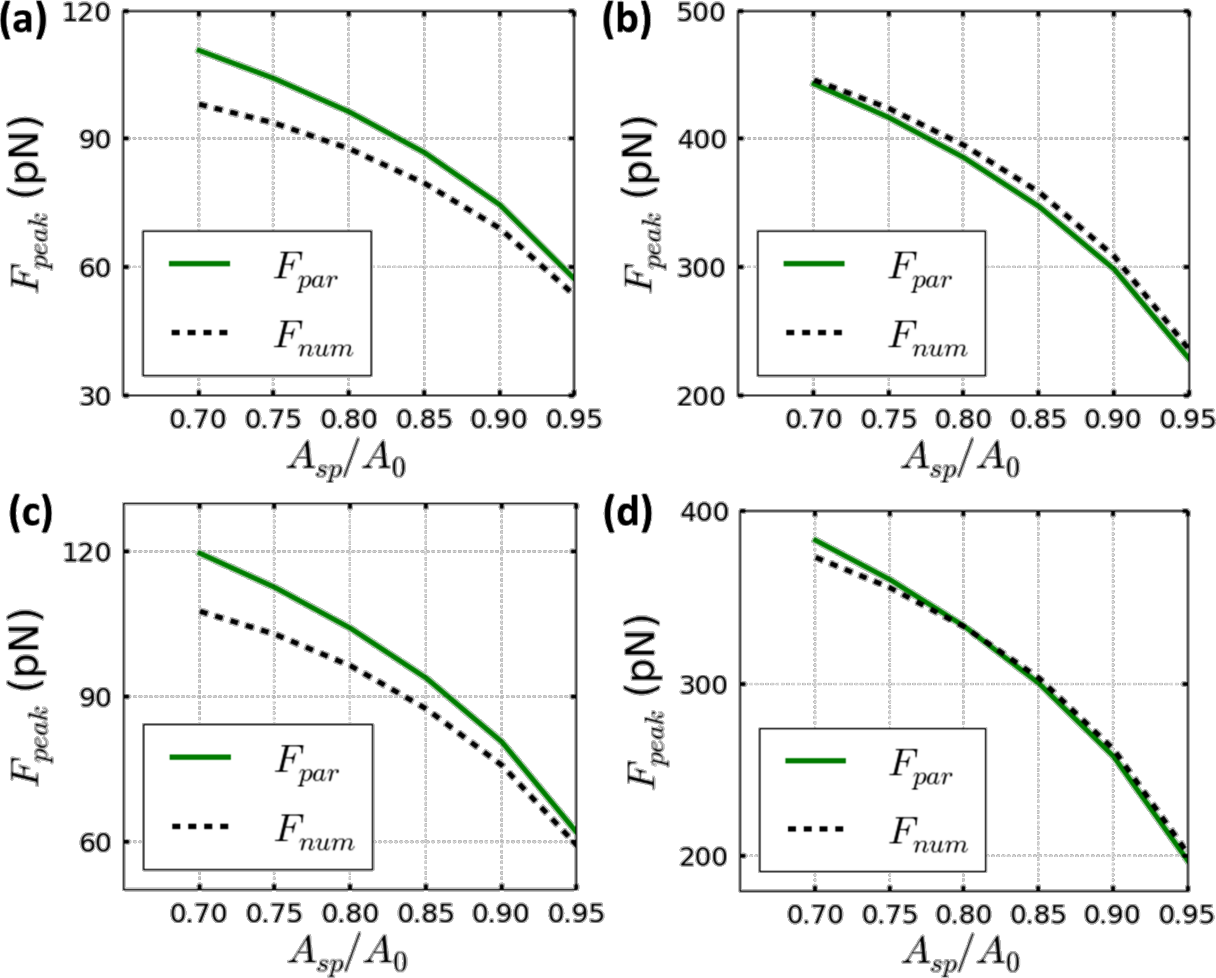
The dependence of the peak forces on the set-point amplitude for the parametrical equation of Equation 8 (full line plots) and the corresponding numerical simulations for Tatara’s contact mechanics (dashed line plots). (a) Material with a Young’s modulus of 200 MPa and *A*_0_ = 1 nm. (b) Material with the same Young’s modulus as (a) and a higher free amplitude *A*_0_ = 4 nm. (c) Material with a Young’s modulus of 300 MPa and *A*_0_ = 1 nm. (d) Material with the same Young’s modulus as (c) and a higher free amplitude *A*_0_ = 4 nm. Parameters are: *Q* = 2, *k* = 0.1 N/m and *R*_t_ = 5 nm.

The dependence of the peak force with *A*_sp_ (from 0.95*A*_0_ to 0.7*A*_0_) is shown in Figure 3 and Figure 4. In general, we have observed that the reduction of *A*_sp_ leads to an increase in the peak force [28–29]. Figure 3 describes the peak force for two materials characterized by a Young’s modulus of 30 MPa (Figure 3a,b) and 100 MPa (Figure 3c,d). The parametrical equation shows a better agreement with the numerical simulations for high *A*_sp_ values. Figure 3a,b shows that the accuracy remains below a relative error of 10% only for set-point amplitudes that do not involve a permanent contact between tip and sample [15,28–29]. The permanent-contact regime depends on the material softness and it does not hold when the Young’s modulus is increased to 100 MPa (Figure 3c,d) within the set-point amplitude values from 0.95*A*_0_ to 0.7*A*_0_.

In addition, good agreement between numerical simulations and Equation 8 can be generally obtained for the range of set-point amplitude values even below 0.7*A*_0_ by maintaining a relative error of 10% (Figure 4b,d). However, it is important to note that the reduction of *A*_sp_ has been halted to 0.7*A*_0_. Smaller set-point values (*A*_sp_ ≈ 0.7*A*_0_) in materials with a Young’s modulus below 60 MPa could imply a permanent-contact regime, which increases the peak interaction force and could lead to a permanent damage of the sample surface [28–29], in particular when the quantitative imaging process involves only elastic mechanical modeling.

The dependence of the peak forces with the microcantilever spring constant follows a power-law dependence that monotonically increases by increasing the value of *k* as shown in previous publications [28–29]. It is important to remark that by increasing *k,* the multi-parametric configurational space for a non-invasive operational regime could be hindered. Hence, Equation 8 and the available operational parameters besides *k* may require a new multi-parametric configurational space, in other words the dependence of the force is proportional to *f*(*E*_eff_, *R*_eff_, *Q*, *k*, *A*_0_, *A*_sp_).

## Conclusion

In short, we have deduced a closed-form equation that rapidly reproduces the peak force exerted by the AFM tip while imaging finite soft materials in liquid. The accuracy of this equation has been verified by means of numerical simulations for archetypical soft materials imaging conditions in AM-AFM based on Tatara’s contact mechanics. Those conditions are oscillation amplitudes in the range of 1–10 nm, and high set-point amplitudes (above 0.8*A*_0_). According to the Young’s moduli of the materials the agreement between the parametrical equation and the numerical simulations remain within a relative error of 10%. However, the accuracy of the present equation decreases when the set-point amplitude value is reduced below 0.8*A*_0_, in particular for soft materials with a Young modulus below 60 MPa. The parametrical equation proposed here extends the quantitative understanding of exerted forces by the tip while imaging soft and elastic materials in liquid environment. It is useful to avoid sample damage while imaging soft materials in liquid with tapping-mode AFM by providing a multi-parametric configurational space. Furthermore this paper provides a new method to deduce parametrical equations applied to dynamic AFM, which can be rapidly extended to further elastic models or different operational parameters.
